# The antagonistic modulation of Arp2/3 activity by N-WASP, WAVE2 and PICK1 defines dynamic changes in astrocyte morphology

**DOI:** 10.1242/jcs.125146

**Published:** 2013-09-01

**Authors:** Kai Murk, Elena M. Blanco Suarez, Louisa M. R. Cockbill, Paul Banks, Jonathan G. Hanley

**Affiliations:** 1 School of Biochemistry, Medical Sciences Building, University of Bristol, Bristol BS8 1TD, UK; 2 School of Physiology and Pharmacology, Medical Sciences Building, University of Bristol, Bristol BS8 1TD, UK

**Keywords:** Actin dynamics, Arp2/3, Astrocyte, Central nervous system, CNS, Morphology, Brain injury

## Abstract

Astrocytes exhibit a complex, branched morphology, allowing them to functionally interact with numerous blood vessels, neighboring glial processes and neuronal elements, including synapses. They also respond to central nervous system (CNS) injury by a process known as astrogliosis, which involves morphological changes, including cell body hypertrophy and thickening of major processes. Following severe injury, astrocytes exhibit drastically reduced morphological complexity and collectively form a glial scar. The mechanistic details behind these morphological changes are unknown. Here, we investigate the regulation of the actin-nucleating Arp2/3 complex in controlling dynamic changes in astrocyte morphology. In contrast to other cell types, Arp2/3 inhibition drives the rapid expansion of astrocyte cell bodies and major processes. This intervention results in a reduced morphological complexity of astrocytes in both dissociated culture and in brain slices. We show that this expansion requires functional myosin II downstream of ROCK and RhoA. Knockdown of the Arp2/3 subunit Arp3 or the Arp2/3 activator N-WASP by siRNA also results in cell body expansion and reduced morphological complexity, whereas depleting WAVE2 specifically reduces the branching complexity of astrocyte processes. By contrast, knockdown of the Arp2/3 inhibitor PICK1 increases astrocyte branching complexity. Furthermore, astrocyte expansion induced by ischemic conditions is delayed by PICK1 knockdown or N-WASP overexpression. Our findings identify a new morphological outcome for Arp2/3 activation in restricting rather than promoting outwards movement of the plasma membrane in astrocytes. The Arp2/3 regulators PICK1, and N-WASP and WAVE2 function antagonistically to control the complexity of astrocyte branched morphology, and this mechanism underlies the morphological changes seen in astrocytes during their response to pathological insult.

## Introduction

Astrocytes are the most abundant glial cell type in the central nervous system (CNS) and have multiple supportive and regulatory roles in neuronal function (Sofroniew and Vinters, 2010). They are probably best known for their homeostatic role, but recent studies have demonstrated an active role of astrocytes in regulating synaptogenesis, synaptic transmission and plasticity through their physical interactions with neurons and specifically synapses (Nedergaard and Verkhratsky, 2012). Each astrocyte possesses its own domain in the CNS and contacts surrounding synapses through a complex network of branched processes. In addition to their functions in the healthy CNS, astrocytes respond to most pathological conditions, including ischemia, with dramatic morphological changes in a process known as reactive astrogliosis. The first signs of astrogliosis are an increase in cell body size and a thickening of the major processes, and, upon severe injuries, astrocytes lose their ‘stellate’ morphology and form physical barriers, known as glial scars. These have beneficial short-term effects by preventing the spread of inflammation and pathogens into the surrounding tissue. However, the glial scar is permanent and inhibits neuronal regeneration (Robel et al., 2011). The actin-based mechanisms that underlie these drastic changes in morphology in response to pathological insult are unknown, and an increase in knowledge of these mechanisms is crucial to a better understanding of neurological disease processes.

A major modulator of actin dynamics is the Arp2/3 complex, which initiates polymerization of new actin filaments (Pollard and Cooper, 2009). Current models on cell motility propose a role for Arp2/3 in pushing the lamellipodial plasma membrane forwards by forming branched actin arrays (Pollard and Cooper, 2009). In accordance with this model, Arp2/3 inactivation leads to impaired cell motility and shrinkage or outgrowth inhibition. The isolated Arp2/3 complex is inactive and must be activated by specific nucleation-promoting factors (NPFs) (Pollard and Cooper, 2009). Within the growing lists of NPFs, the WAVE complex and N-WASP are the best characterized. The WAVE complex is crucial for lamellipodia formation and membrane ruffling, whereas numerous studies indicate a prominent role for N-WASP in endocytosis and vesicle motility (Rottner and Stradal, 2011). To date, there are no reports describing the function of WAVE or N-WASP in astrocytes. Endogenous Arp2/3 inhibitor proteins include PICK1, which inhibits actin nucleation through direct binding to the Arp2/3 complex (Rocca et al., 2008; Nakamura et al., 2011). PICK1-mediated inhibition of Arp2/3-mediated actin polymerization has been demonstrated in neurons, but its role in regulating actin dynamics in other cell types is unexplored.

In the present study, we show that tonic Arp2/3 activity is required for maintaining the typical stellate morphology of astrocytes in dissociated cultures and in brain tissue. In striking contrast to other cell types, Arp2/3 inhibition evokes cell body expansion in astrocytes. Furthermore, we identify N-WASP, WAVE2 and PICK1 as antagonistic endogenous Arp2/3 regulators involved in the regulation of astrocyte morphology, particularly under conditions relating to ischemic injury.

## Results

### Inactivation of the Arp2/3 complex evokes cell expansion in astrocytes

To study the role of the Arp2/3 complex in astrocytes, we used the compound CK-548, which specifically blocks Arp2/3 dependent actin nucleation and has been successfully used on monocytes and neurons (Nolen et al., 2009; Tahirovic et al., 2010).

**Fig. 1.**
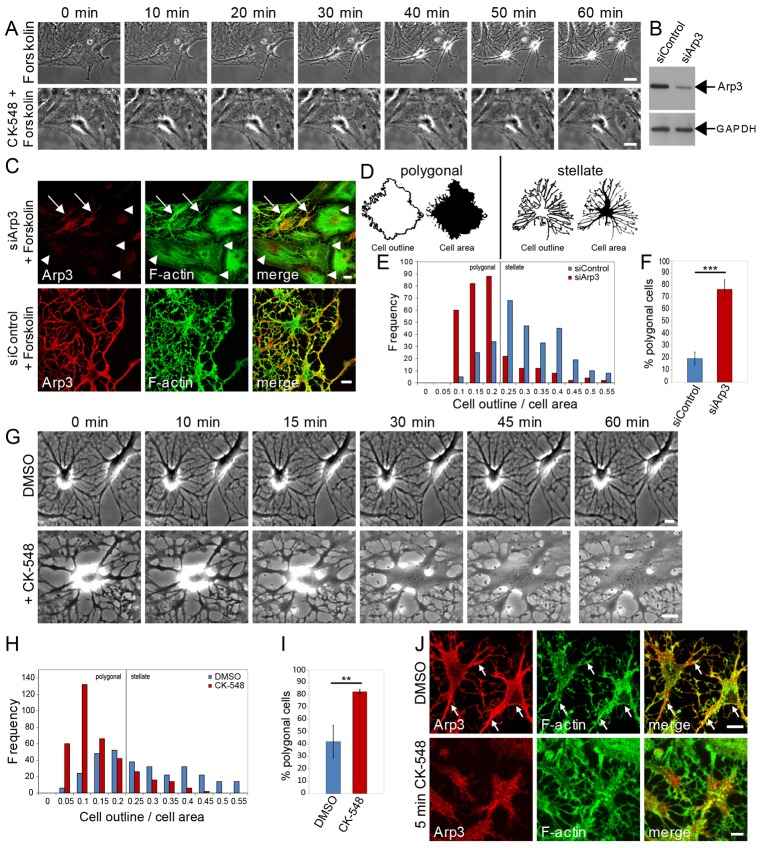
**Inactivation of the Arp2/3 complex in astrocytes results in an expanded destellated morphology.** (**A**) Phase-contrast live-cell imaging of serum-starved cultured astrocytes in the presence of forskolin with or without the Arp2/3 inhibitor CK-548. Scale bars: 20 µm. (**B**) Western blot analysis of astrocytes transfected either with a control (siControl) or Arp3 (siArp3)-specific siRNA. Arp3 expression was determined using an Arp3-specific antibody, and GAPDH immunoreactivity was used as loading control. (**C**) Confocal images of astrocytes transfected with Arp3 siRNA or control siRNA followed by serum starvation and forskolin treatment. Arp3 expression was visualized by immunostaining for Arp3 (red) and F-actin by phalloidin staining (green). Arp3-depleted cells (arrowheads) do not have a stellated morphology, compared with Arp3-positive cells (arrows), which do. Scale bars: 10 µm. (**D**) Schematic example to illustrate representative differences in cell outlines and cell areas of polygonal (left) and stellate (right) astrocytes. (**E**) Frequency analysis of astrocyte complexity in Arp3-knockdown and control cells after forskolin treatment. Cells were analyzed regarding the ratio of cell outline and cell area. Cells with a cell outline to cell area ≤0.2 are defined as polygonal. High values for the cell-outline:cell-area ratios correspond to high levels of astrocyte complexity (*n* = 300 cells per condition from three independent experiments). (**F**) Quantification of the proportion of polygonal cells as shown in C and E. ***P<0.0005 (unpaired Student's *t*-test). (**G**) Phase contrast live-cell imaging of serum-starved astrocytes previously treated with forskolin and kept in serum-free medium in the absence or presence of CK-548. Scale bars: 10 µm. (**H**) Frequency analysis of astrocyte complexity of CK-548- and DMSO-treated cells (300 cells per condition from three independent experiments). (**I**) Quantification of the proportion of polygonal cells shown in G and I. **P<0.005 (Student's unpaired *t*-test). (**J**) Confocal images of stellated astrocytes before (upper panels), after 5 min of CK-548 incubation. Arp2/3 localization and actin filaments were visualized by immunostaining for Arp3 (red) and phalloidin staining for F-actin (green). Note that Arp3 is enriched along processes and the plasma membrane of stellated astrocytes (arrows). Scale bars: 10 µm.

Rat astrocytes grow as polygonal cells in serum-rich medium, so we used a previously established protocol to drive astrocytes into a stellate morphology analogous to astrocytes in native tissue (Ramakers and Moolenaar, 1998). Forskolin stimulates actin bundle disassembly, cytoplasmic shrinkage and process outgrowth (Fig. 1A; supplementary material Movie 1). These morphological changes are completely blocked by CK-548 application (Fig. 1A; supplementary material Movie 2).

To further analyze the role of Arp2/3 activity in regulating astrocyte morphology, we transfected astrocytes with a previously verified Arp3-specific siRNA (Korobova and Svitkina, 2008). Five days post-transfection, Arp3 expression was reduced to 30.2%±4.8 (s.d.) compared with that in controls (Fig. 1B). In Arp3-deficient astrocytes, forskolin-induced stellation was completely blocked (Fig. 1C, arrowheads), whereas cells with higher Arp3 expression acquire a stellated morphology (Fig. 1C, arrows). To quantify the morphological changes, we established a method to distinguish between cell shapes ranging from polygonal to stellate astrocyte morphologies. Higher values of the ratio between the cell outline and the cell area indicate greater astrocyte complexity, that is a greater degree of stellation, whereas cells with values ranging from 0 to 0.2 have a polygonal shape (Fig. 1D). Arp3 knockdown blocks the development of the typical stellate astrocyte morphology in 76.6%±7.8 of cells, whereas under control conditions only 19.5%±5.3 of astrocytes fail to respond to forskolin (Fig. 1E,F). Arp3 knockdown therefore resembles the phenotype observed with the pharmacological inhibitor CK-548.

To investigate the effect of Arp2/3 inhibition on previously stellated astrocytes, we treated astrocytes with forskolin, and after washout incubated with CK-548. In the absence of any other extrinsic signal, Arp2/3 inhibition evokes the expansion of astrocytes towards a polygonal morphology (Fig. 1G; supplementary material Movie 4). DMSO-treated control cells retain their stellate morphology (Fig. 1G; supplementary material Movie 3). The changes in astrocyte morphology triggered by Arp2/3 inhibition were largely complete within 45–60 min, and 76%±3.7 of cells were defined as being polygonal after 2 h of CK-548 incubation (Fig. 1H,I). Arp2/3 inhibition by CK-548 also caused rapid changes in Arp2/3 localization. Instead of being enriched along processes and the plasma membrane, the Arp2/3 complex had a scattered localization immediately after its inactivation by CK-548 (Fig. 1J). To investigate whether the unexpected effect of Arp2/3 inhibition reflects a different protein expression level of the Arp2/3 complex compared with that in other cell types, we analyzed the expression level of the key subunit Arp3 in astrocytes, other primary cells and two cell lines including a cancer cell line (supplementary material Fig. S1). We did not detect any significant differences in Arp3 levels in astrocytes compared with that in neurons or primary embryonic fibroblasts (supplementary material Fig. S1A,B). In summary, these results demonstrate an unexpected expansion of cultured astrocytes as a consequence of Arp2/3 inhibition.

**Fig. 2.**
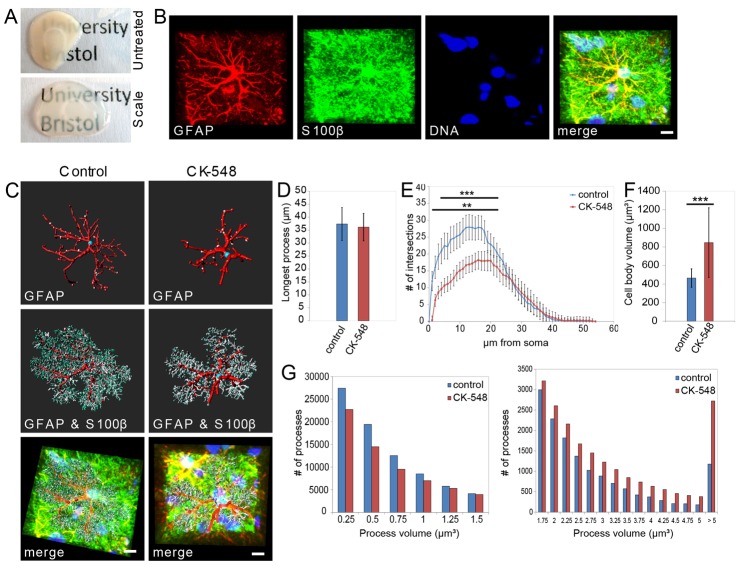
**Acute inhibition of the Arp2/3 complex in astrocytes in brain slices.** (**A**) Demonstration of a modified tissue clearance procedure, allowing deep-tissue antibody staining and imaging by confocal microscopy. Untreated cortical slice (top) in comparison to cleared tissue (bottom). (**B**) *Z*-projection of a control astrocyte 40 µm within the cortical slice, previously stained for DNA (Hoechst 33258), GFAP and S100β, as acquired by confocal microscopy after tissue clearance. Scale bars: 10 µm. (**C**) Filament tracing of GFAP- and S100β-positive processes in an individual astrocyte. Main processes were defined by GFAP immunoreactivity (top). Fine structures were determined by S100β immunoreactivity (center). Overlay of 3D models and confocal *z*-projections (bottom). Left panels: control astrocyte. Right panels: astrocyte from a slice treated with CK-548. Scale bars: 10 µm. (**D**) Quantification of longest processes in control and CK-548-treated astrocytes, based on GFAP immunoreactivity. *n* = 20 per condition, *P*>0.05 (unpaired Student's *t*-test). (**E**) Sholl analyzes on combined GFAP- and S100β-positive processes in control (blue) and CK-548-treated (red) astrocytes. *n* = 20 per condition, **P<0.005, ***P<0.0005 (unpaired Student's *t*-test and Sidak–Bonferroni method). (**F**) Quantification of soma volumes from control and CK-548-treated astrocytes. *n* = 20 per condition, ***P<0.0005 (unpaired Student's *t*-test). (**G**) Frequency of individual S100β-positive process volumes from control and CK-548-treated astrocytes. (90,000 S100β-positive processes from 20 cells per condition). Left graph: small processes up to 1.25 µm^3^. Right graph: larger processes greater than 1.5 µm^3^.

To study whether Arp2/3 inhibition would evoke analogous changes in astrocytes in intact tissue, we exposed acute cortical slices from P14 rats to CK-548 (Fig. 2). To detect changes in astrocyte morphology within tissue, we established a new technique to combine whole-mount immunohistochemistry with a recently published method for tissue clearance (see Materials and Methods) (Fig. 2A) (Hama et al., 2011), allowing antibody labeling for astrocytic markers observable deep within the tissue by confocal microscopy (supplementary material Movie 5). We used antibodies specific for glial fibrillary acidic protein (GFAP), which labels the main astrocytic processes, and S100β, which localizes to the cytoplasm and, to a minor extent, to membranes (Sen and Belli, 2007) (Fig. 2B). S100β staining therefore acts as a useful marker to define the fine details of complex astrocyte morphology (Fig. 2B, supplementary material Movie 6). We used a filament-tracing algorithm in Imaris software, originally designed for quantifying dendritic spines on neurons. We defined GFAP-positive processes as ‘dendrites’ from where smaller processes (‘spines’) originate and branch (Fig. 2C). To study the effect of Arp2/3 inhibition on astrocytes, we incubated cortical slices with either CK-548 or DMSO in oxygenated artificial cerebrospinal fluid (aCSF) and performed quantitative analyzes of astrocyte morphology. Fig. 2D shows that CK-548 has no effect on the length of the longest GFAP-positive process. Astrocytes from control slices exhibit a highly complex arborization of fine, branched S100β-positive processes emerging from the main shafts. In contrast, astrocytes in CK-548-treated slices show fewer, thicker S100β-containing protrusions compared with the number in control cells (Fig. 2C). To quantify these changes in astrocyte complexity, we performed Sholl analyzes taking GFAP- as well as S100β-positive processes into account. Pharmacological inhibition of the Arp2/3 complex leads to a dramatic reduction in astrocyte complexity compared with that in control cells (Fig. 2E). In addition, we studied whether the volume of individual S100β-containing protrusions changes in response to Arp2/3 inhibition. We observed a decrease in the number of small protrusions, whereas bigger processes become more frequent (Fig. 2G). Furthermore, inhibition of Arp2/3 activity causes a marked increase in cell body volume (Fig. 2F). These experiments demonstrate that acute inhibition of the Arp2/3 complex also evokes the expansion of astrocytes within intact tissue.

### Myosin II and formins drive the cell expansion of astrocytes during Arp2/3 inhibition

**Fig. 3.**
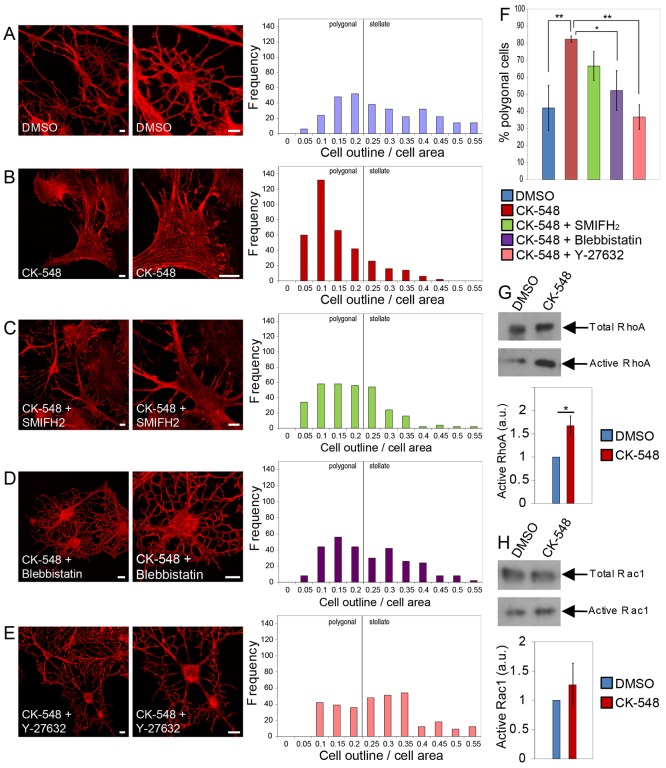
**Inhibition of formins and Myosin II counteracts Arp2/3 inhibition and is associated with changes in small GTPase activation.** Images (left) and frequency analysis (right) for astrocytes after serum starvation, forskolin and subsequent incubation with DMSO (**A**), CK-548 (**B**), CK-548 plus the formin inhibitor SMIFH2 (**C**), CK-548 plus blebbistatin (**D**) and CK-548 plus the ROCK inhibitor Y-27632 (**E**). Scale bars: 10 µm. Cell morphology is visualized by Alexa-546–phalloidin staining. Graphs show quantification of astrocyte morphology following the drug treatments (*n* = 300 cells per condition from three independent experiments). (**F**) Quantification of the proportion of polygonal astrocytes after the treatments shown in A–E. *P<0.05, **P<0.005 (ANOVA followed by Bonferroni's correction). (**G**) Determination of RhoA activation in astrocytes after forskolin treatment followed by CK-548 treatment for 1 h. Upper blots indicate total RhoA levels, lower blots show the GTP-bound fraction. The graph shows a quantification of the relative proportion of active RhoA, as shown in the western blots. *n* = 5, *P<0.05 (unpaired Student's *t*-test). (**H**) Determination of Rac1 activation in astrocytes after forskolin treatment, followed by CK-548 treatment for 1 h. Upper blots indicate total Rac1 levels, lower blots show the GTP-bound fraction. The graph shows a quantification of the relative proportion of active Rac1, as shown in the western blots. *n* = 4. *P>0.05 (unpaired Student's *t*-test).

To investigate the molecular mechanisms responsible for the Arp2/3-dependent expansion of astrocytes, we incubated stellated astrocyte cultures with CK-548 in conjunction with small inhibitors specific for other cytoskeletal components (Fig. 3A–E). Cells treated with CK-548 or DMSO alone served as controls (Fig. 3A,B). The formin inhibitor SMIFH2 (Rizvi et al., 2009) appeared to cause a small reduction in the effect of CK-548 on cell expansion (Fig. 3C). However, statistical analysis indicated that there was no significant difference in the proportion of polygonal cells in astrocytes treated with CK-548 and SMIFH2 compared with in astrocytes treated with CK-548 alone (Fig. 3F). The myosin II inhibitor blebbistatin more completely blocked astrocyte expansion (Fig. 3D,F). This result led us to test upstream regulators of myosin II in the presence of CK-548. We used Y-27632 to inhibit the Rho-dependent kinase (ROCK), which activates myosin II (Vicente-Manzanares et al., 2009). The efficiency of Y-27632 in counteracting the effects of Arp2/3 inhibition on astrocyte morphology is similar to that of blebbistatin (Fig. 3D–F).

The opposing effect of the inhibitor Y-27632- on CK-548-treated astrocytes suggests changes in upstream signaling pathways mediated by the small GTPase RhoA. We performed pulldown assays to isolate GTP-bound GTPases from astrocytes treated with either DMSO or CK-548 after forskolin washout. In comparison with control cells, the level of GTP-bound RhoA is significantly increased in CK-548-treated astrocytes (Fig. 3G). We also measured the amount of active Rac1 in the treated astrocytes. In contrast to RhoA, no significant changes occurred in the level of GTP-bound Rac1 (Fig. 3H). These results indicate that the expansion of astrocytes is accompanied with increased RhoA activity.

### N-WASP is a crucial Arp2/3 activator in astrocyte morphology

To investigate which endogenous Arp2/3 regulatory proteins are required for the maintenance of astrocyte morphology, we knocked down expression of the Arp2/3 activators N-WASP and WAVE. A previous study demonstrated that astrocytes exclusively express the ubiquitous WAVE2 isoform, with undetectable levels of WAVE1 and WAVE3 (Kim et al., 2006).

**Fig. 4.**
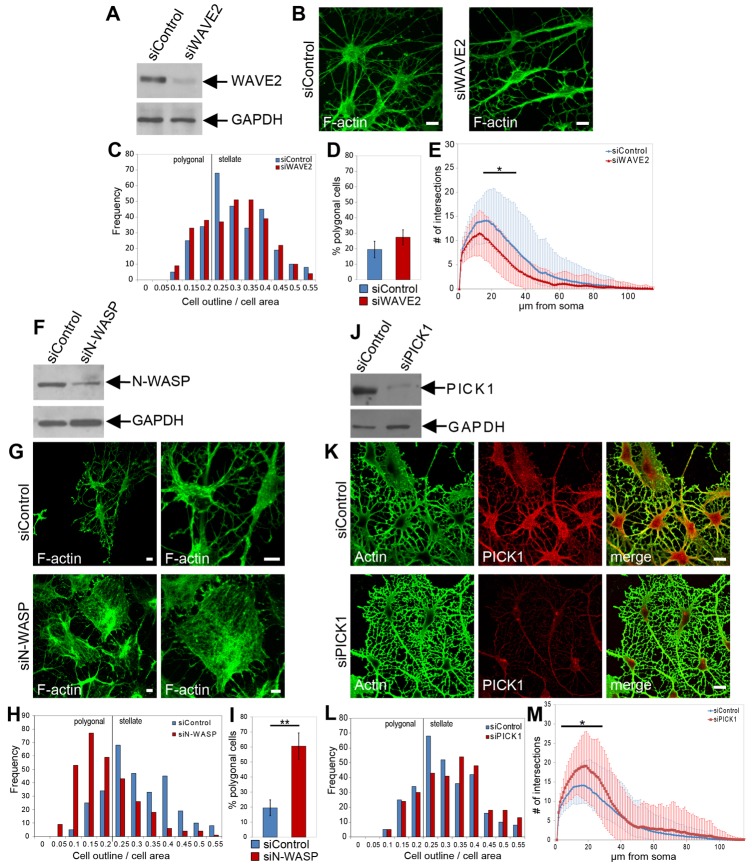
**Identification of Arp2/3 regulators in astrocytes.** (**A**) Astrocytes were transfected with control siRNA (siControl) or WAVE2-specific siRNA (siWAVE2). WAVE2 and GAPDH expression were analyzed by western blotting. (**B**) Confocal images of siControl- and siWAVE2-transfected astrocytes, subjected to by serum starvation, forskolin treatment and phalloidin staining. Scale bars: 10 µm. (**C**) Frequency analysis of the astrocyte complexity in WAVE2-knockdown and control cells after forskolin treatment. For frequency analysis, *n* = 300 cells per condition from three independent experiments. (**D**) Quantification of the proportion of polygonal astrocytes of siControl- and siWAVE2-transfected cells, *P<0.05 (unpaired *t*-test). (**E**) Sholl analysis on processes of astrocytes transfected with either control siRNA (siControl, blue) or siWAVE2-specific siRNA (siWAVE2, red). *n* = 108 (siControl), *n* = 140 (siWAVE2), *P<0.05 (unpaired *t*-test and Sidak-Bonferroni method). (**F**) Astrocytes were transfected with control siRNA (siControl) or N-WASP-specific siRNA (siN-WASP). N-WASP and GAPDH expression were analyzed by western blotting. (**G**) Confocal images of astrocytes after transfection with control siRNA (siControl) or N-WASP-specific siRNA (siN-WASP), followed by serum starvation, forskolin treatment and phalloidin staining. Scale bars: 10 µm. (**H**) Frequency analysis of astrocyte complexity of N-WASP-knockdown and control cells after forskolin treatment. For frequency analysis, *n* = 300 cells per condition from three independent experiments. (**I**) Quantification of the proportion of polygonal astrocytes from control (siControl) and N-WASP-depleted cells (siN-WASP), as shown in H. **P<0.005 (unpaired Student's *t*-test). (**J**) Astrocytes were transfected with control siRNA (siControl) or PICK1-specific siRNAs (siPICK1). PICK1 and GAPDH expression were analyzed by western blotting. (**K**) Confocal images of astrocytes after transfection with control siRNA (left) and PICK1-specific siRNA (right), stained with actin and PICK1-specific antibodies. Before fixation and immunocytochemistry, cells were serum-starved and treated with forskolin. Scale bars: 10 µm. (**L**) Frequency analysis of astrocyte complexity of PICK1-knockdown and control cells after forskolin treatment (300 cells per condition from three independent experiments in each frequency analysis). (**M**) Sholl analysis on processes of astrocytes transfected with either control siRNA (siControl, blue) or PICK1-specific siRNA (siPICK1, red). *n* = 108 (siControl), *n* = 82 (siPICK1), *P<0.05 (unpaired Student's *t*-test and Sidak–Bonferroni method).

We used a published siRNA sequence (Danson et al., 2007), which reduces endogenous WAVE2 expression to 27.4%±7.2 in cultured astrocytes (Fig. 4A). Most WAVE2-depleted cells acquired a stellate morphology following forskolin treatment, and the proportion of polygonal cells was not significantly changed from controls (Fig. 4B–D). To further investigate whether the depletion of WAVE2 affected astrocyte arborization, we employed Sholl analyzes, and specifically analyzed astrocytic processes of stellate astrocytes (with a cell-outline:cell-area ratio greater than 0.2) not including cell bodies. Despite a high variance, we were able to determine a significantly decreased complexity in WAVE2-depleted astrocytes (Fig. 4E). These findings demonstrate that WAVE2 is involved in organizing astrocytic processes, however knocking down WAVE2 did not completely block changes in astrocyte morphology, as observed upon Arp2/3 inhibition (see Fig. 1).

We carried out similar experiments to examine the role of N-WASP. A previously characterized siRNA (Kovacs et al., 2011) reduced endogenous N-WASP to 33%±2.4 of its normal level in astrocytes (Fig. 4F). In contrast to WAVE2-deficient cells, astrocytes with reduced N-WASP expression showed a complete block of stellation, and remained in a polygonal morphology (Fig. 4G–I). This demonstrates that N-WASP is required for the development of the typical astrocyte morphology, and strongly suggests that N-WASP is the major Arp2/3 activator in this process.

### Depletion of the Arp2/3 inhibitor PICK1 leads to increased astrocyte complexity

Given that astrogliosis involves cell body expansion and reduced astrocyte complexity, and our data demonstrate a role for Arp2/3 inhibition in this process, we investigated the role of an endogenous Arp2/3 inhibitory protein that might function antagonistically to N-WASP. We previously defined PICK1 as an Arp2/3 inhibitor that can oppose N-WASP in actin polymerization assays (Rocca et al., 2008). To manipulate PICK1 levels in astrocytes, we used a recently published shRNA sequence (Citri et al., 2010) to design synthetic siRNA, which depletes endogenous PICK1 in astrocytes to 6%±4 of control levels (Fig. 4J). Showing the opposite effect to depletion of N-WASP or Arp3, PICK1 knockdown results in increased branching of astrocytic processes after forskolin treatment (Fig. 4K–M). Because this treatment leads to increased complexity, there is no significant change in the proportion of polygonal astrocytes following PICK1 knockdown (supplementary material Fig. S2). PICK1 depletion does not affect cell morphology or actin organization in polygonal astrocytes that were not treated with forskolin (supplementary material Fig. S3A).

Taken together, these experiments demonstrate a role for N-WASP and WAVE2 as Arp2/3 activators, and PICK1 as an Arp2/3 inhibitor with opposing roles in regulating astrocyte morphology.

### Knockdown of PICK1 inhibits morphological changes in astrocytes following oxygen and glucose deprivation

**Fig. 5.**
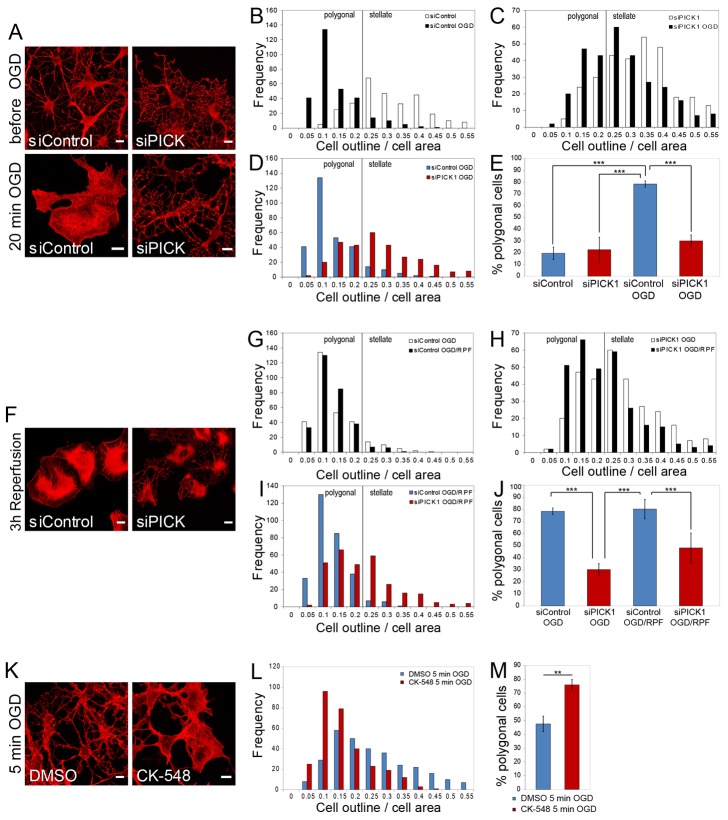
**PICK1 knockdown inhibits morphological changes in astrocytes in response to OGD.** (**A**) Confocal images of serum-starved and forskolin-treated astrocytes before and after 20 min of OGD. Cell morphology was visualized by F-actin staining with phalloidin–Alexa-546. Scale bars: 10 µm. (**B**) Frequency analysis on complexity of control astrocytes before and after 20 min OGD (*n* = 300 cells per condition from three independent experiments for each frequency analysis). (**C**) Frequency analysis on cell complexity of PICK1-deficient astrocytes before and after 20 min OGD (*n* = 300 cells per condition from three independent experiments for each frequency analysis). (**D**) Direct comparison of cell complexities of control and PICK1-deficient astrocytes after 20 min OGD. (**E**) Quantification of the proportion of polygonal astrocytes, as shown in B, C and D. ***P<0.0005 (ANOVA with Bonferroni's correction). (**F**) Confocal images of serum-starved and forskolin-treated siControl- and siPICK1-transfected astrocytes after 20 min OGD, followed by 3 h reperfusion with oxygenated and glucose-containing basal medium. Visualization of morphology by F-actin staining with phalloidin–Alexa-546. Scale bars: 10 µm. (**G**) Frequency analysis on cell complexity of control astrocytes after 20 min OGD, and after 20 min OGD and 3 h of reperfusion (OGD/RPF) (*n* = 300 cells per condition from three independent experiments for each frequency analysis). (**H**) Frequency analysis on cell complexity of PICK1-deficient astrocytes after 20 min OGD, and after 20 min OGD and 3 h of reperfusion of reperfusion (OGD/RPF) (*n* = 300 cells per condition from three independent experiments for each frequency analysis). (**I**) Frequency analysis on cell complexity of control and PICK1-deficient astrocytes after 20 min OGD and 3 h of reperfusion (OGD/RPF). (**J**) Quantification of the proportion of polygonal astrocytes, as shown in G, H and I. ***P<0.0005 (ANOVA with Bonferroni's correction). (**K**) Confocal images of serum-starved and forskolin-treated astrocytes after 5 min of OGD with either DMSO or CK-548. Cell morphology was visualized by F-actin staining with phalloidin–Alexa-546. Scale bars: 10 µm. (**L**) Frequency analysis of astrocytes after 5 min OGD and treated with either DMSO or CK-548 (*n* = 300 cells per condition from three independent experiments for each frequency analysis). (**M**) Quantification of the proportion of polygonal astrocytes, as shown in L. **P<0.0005 (unpaired Student's *t*-test).

To directly test whether the manipulation of the Arp2/3-complex machinery can influence injury-associated changes in astrocyte morphology, we knocked down PICK1 using siRNA and exposed these cells to oxygen and glucose deprivation (OGD), an *in vitro* model for ischemia. Within 20 min of OGD, control astrocytes completely lose their typical stellated astrocyte morphology and acquire a polygonal cell morphology, which is accompanied by a substantial increase in visible actin fibers (Fig. 5A,B,D,E). Interestingly, PICK1-depleted astrocytes exhibit dramatically reduced OGD-dependent astrocyte expansion (Fig. 5A,C,D,E), strongly suggesting that PICK1 is required for injury-associated changes in astrocyte morphology that occur during astrogliosis.

After cerebral ischemia, brain cells undergo so-called reperfusion injuries caused by free radicals when the blood circulation has been restored (Alexandrov 2010). We simulated this condition after OGD by reperfusing cultured cells with oxygenated glucose-containing medium for 3 h, which evokes further formation of thick actin fibers (Fig. 5F). There is no further change in overall morphology (Fig. 5G). In contrast, a substantial subset of PICK1-depleted astrocytes do not acquire a complete polygonal cell shape and still exhibit slender processes even after 3 h of reperfusion (Fig. 5F,H–J). Although the remaining PICK1-deficient cells acquire a polygonal shape after reperfusion, these astrocytes do not show the same level of cell expansion and actin fiber formation as control cells after reperfusion (Fig. 5F). These results suggest that Arp2/3 inhibition by PICK1 leads to astrocyte expansion during OGD. To directly test whether OGD-induced astrocyte expansion requires Arp2/3 inactivation, we performed a ‘sub-threshold’ OGD experiment. A 5-min OGD treatment has only minor effects on astrocyte morphology (Fig. 5K–M), and CK-548 alone also has no detectable effect within 5 min (Fig. 1G). In contrast, the addition of CK-548 leads to a rapid increase in the number of polygonal astrocytes within 5 min of OGD (Fig. 5K–M).

### Arp2/3 overactivation by N-WASP blocks astrocyte expansion during OGD

**Fig. 6.**
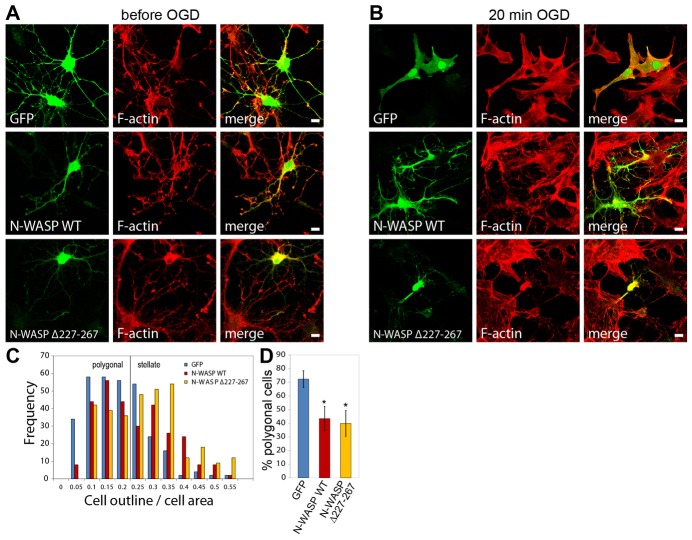
**Arp2/3 stimulation by N-WASP overexpression inhibits OGD-dependent changes in astrocyte morphology.** Astrocytes were transfected with GFP, GFP–N-WASP WT or constitutively active GFP–N-WASP-Δ227–267. (**A**) Confocal images of transfected, serum-starved and forskolin-treated astrocytes before OGD, stained with phalloidin–Alexa-546. Scale bars: 10 µm. (**B**) Confocal images of transfected astrocytes after 20 min OGD and stained for F-actin. Scale bars: 10 µm. (**C**) Frequency analysis of complexity of astrocytes transfected with GFP, N-WASP WT and N-WASP-Δ227–267 after OGD. For this analysis only cells with exogenous N-WASP in the cytosol and nuclei were taken into account (300 cells per condition from three independent experiments were used in each frequency analysis). (**D**) Quantification of the proportion of polygonal astrocytes, as shown in C. *P<0.05 (unpaired Student's *t*-test).

To further explore the mechanism behind OGD-induced astrocyte expansion, we overexpressed GFP-tagged N-WASP variants. Compared with control cells transfected with GFP, N-WASP overexpressing astrocytes exhibit fewer stress fibers (supplementary material Fig. S3B). Moreover, a constitutively active mutant of N-WASP, Δ226-267 (Stamm et al., 2005), radically alters F-actin organization in polygonal astrocytes (supplementary material Fig. S3B). We assume that the differences in actin organization between astrocytes expressing N-WASP WT and N-WASP Δ226–267 are based on the fact that the activity of overexpressed N-WASP WT relies on upstream signaling pathways present in the transfected astrocytes. All transfected cells expressing either GFP or N-WASP variants acquire a stellate morphology by forskolin treatment (Fig. 6A). During OGD, 72.3%±6.1 of GFP-expressing control cells expand to a polygonal morphology (Fig. 6B–D). In astrocytes overexpressing GFP-tagged wild-type (WT) N-WASP, destellation is markedly inhibited, and only 43.3%±9 of cells exhibit a polygonal morphology after OGD (Fig. 6B–D). Before OGD, all GFP–N-WASP-Δ226–267-expressing astrocytes exhibit a stellate morphology and exhibit a frequency of polygonal cells, analogous to astrocytes overexpressing N-WASP-WT (Fig. 6D). However, stellate N-WASP-Δ226–267-expressing cells tend to be a higher cell-outline:cell-area ratio than astrocytes transfected with N-WASP WT (Fig. 6C).

Taken together, these results demonstrate that the level of Arp2/3 activation, controlled by PICK1 and N-WASP, defines astrocyte morphological complexity under conditions relating to ischemic injury.

## Discussion

In this study, we investigated the role of the Arp2/3 complex and associated signaling in astrocytes. We demonstrate that the expansion of astrocytic cell bodies and processes is triggered by Arp2/3 inhibition in dissociated cultures and brain tissue. This phenomenon requires the activity of myosin II in conjunction with increased RhoA activity. Furthermore, we identified N-WASP and PICK1 as crucial Arp2/3 regulators in astrocyte morphological plasticity, and show that this mechanism underlies the rapid and drastic morphological changes exhibited by astrocytes under ischemic conditions.

In most studied cell types, inactivation of the Arp2/3 complex leads mainly to disappearance, outgrowth-inhibition or shrinkage of subcellular structures such as lamellipodia in fibroblasts and cancer cells (Steffen et al., 2006; Wu et al., 2012), or neurites and dendritic spines in neurons (Korobova and Svitkina, 2008; Hotulainen et al., 2009; Tahirovic et al., 2010; Nakamura et al., 2011; Yang et al., 2012). Acute inactivation of the Arp2/3 complex in astrocytes has the opposite effect, and leads to cell body expansion. This demonstrates that astrocytes employ the actin cytoskeleton in a distinct manner to define their morphology, compared to other cell types. Although astrocytes are capable of acquiring morphologies similar to non-neuronal cells and neurons, our results show that the Arp2/3-dependent mechanisms used for these morphological changes are different in astrocytes compared with other cell types.

Our small-molecule inhibitor and RNAi experiments indicate that astrocytes require a high tonic activation of the Arp2/3 complex through N-WASP or WAVE to obtain and maintain their typical stellate morphology. This suggests that the Arp2/3 complex forms an actin network that provides membrane tension to maintain a complex stellate morphology. Furthermore, our results show that the Arp2/3-dependent expansion of astrocytes to a polygonal morphology can be prevented by the inhibition of myosin II and its upstream activator ROCK, indicating that myosins play a key role during the transition from stellate to polygonal astrocytes. Actin arrays formed by the Arp2/3 complex usually do not contain myosins, which could explain the low level of active myosin in stellate astrocytes, and a higher level in polygonal astrocytes (John et al., 2004; Vicente-Manzanares et al., 2009). This hypothesis is supported by a recent study on the interplay of the Arp2/3 complex and myosin II in neuronal growth cones (Yang et al., 2012). In conjunction with previous *in vitro* experiments (Janson et al., 1991), these studies suggest that Arp2/3 activity creates a dense actin network in the cell cortex, which resists myosin II contractility. A possible mechanism for myosin-dependent astrocyte expansion could be that the loss of Arp2/3-dependent actin networks allows the re-distribution of myosin into the periphery of astrocytes. This is consistent with a previous study reporting that myosin is particularly enriched in the periphery of polygonal astrocytes (John et al., 2004). Within the cell cortex, active myosin could re-organize actin filaments from destabilizing branched arrays towards bundles followed by the assembly of larger focal adhesions (Favero and Mandell, 2007; Vicente-Manzanares et al., 2009). Both actin fiber formation and re-organization of focal adhesions might then consolidate ‘filling the gap’ membrane progression between primary astrocyte processes (supplementary material Movie 4) in a similar manner to a mechanism recently described for Arp2/3-deficient fibroblasts (Wu et al., 2012). However, these protrusions appear in astrocytes in a non-polarized manner and thereby evoke comprehensive cell spreading towards a polygonal morphology. Further research is necessary to study the precise nature of actin networks in astrocytes.

Previously, elevated RhoA activity had been detected in neurons after Arp3 depletion (Korobova and Svitkina, 2008). We also measured increased levels of active RhoA in CK-548-treated astrocytes but observed no significant changes in active Rac1. This coincidence of astrocyte expansion and higher RhoA activity is consistent with previous studies showing that inactivation of RhoA and myosin is necessary for astrocytes to acquire and maintain a stellate morphology (Ramakers and Moolenaar, 1998; John et al., 2004). The precise mechanism as to how increased RhoA activity is triggered in stellate astrocytes upon Arp2/3 inhibition is unknown and requires further investigation. However, we can exclude the possibility of a feedback loop in astrocytes mediated through Rac1 inactivation (Tang et al., 2012), as the levels of active Rac1 are not significantly changed in CK-548-treated astrocytes (Fig. 3H).

Although we occasionally observe increased filopodia formation in astrocytes with inactive Arp2/3 complex (supplementary material Movie 4), our quantification of stellated astrocytes treated with CK-548 and SMIFH2 does not provide evidence for a significant contribution of formins to the transition of stellate astrocytes to polygonal cells (Fig. 3F).

We identify N-WASP as a major Arp2/3 activator that controls overall astrocyte morphology. Consistent with the Arp2/3 inactivation experiments, astrocytes with reduced N-WASP levels show defects in developing a stellate morphology, whereas the knockdown of the only expressed WAVE isoform (WAVE2) affects only the formation of astrocytic processes. These results might indicate a crucial role for N-WASP as Arp2/3 activator for the general astrocyte morphology, whereas WAVE2 modulates the fine organization of astrocytic processes. Analogous to SCAR and WAVE knockouts in *Dicytostelium*, it is also conceivable that, in astrocytes, N-WASP and WAVE2 might share functions in Arp2/3 regulation but that N-WASP compensates for the loss of WAVE2 (Veltman et al., 2012). Conversely, PICK1 knockdown promotes astrocyte complexity and thereby evokes the opposite phenotype to Arp3, N-WASP or WAVE2 depletion. Antagonism between PICK1 and N-WASP has been observed before in *in vitro* actin polymerization assays (Rocca et al., 2008), but not in cell physiology. The importance of the opposing roles of PICK1 and N-WASP *in vivo* is highlighted in situations of ischemic injury. We analyzed the effect of OGD on stellated astrocytes, which exhibit rapid changes in morphology during brief exposures to ischemic conditions. Overactivating the Arp2/3 complex by PICK1 depletion or N-WASP overexpression inhibits OGD-induced morphological changes. In contrast, pharmacological Arp2/3 inhibition during OGD rapidly accelerates (within a few minutes) the expansion of astrocytes from stellate to polygonal cells. Our results suggest that there could be a switch in Arp2/3 activity within astrocytes during ischemia. In the healthy CNS, high levels of active N-WASP provide a tonic activation of the Arp2/3 complex, and thereby maintain the typical astrocyte morphological complexity. During ischemia, the Arp2/3 complex is inhibited by PICK1, resulting in the previously described mechanism for astrocyte expansion. This hypothesis is supported by the recent description of elevated levels of PICK1 in reactive astrocytes in an animal model of amyotrophic lateral sclerosis (ALS) (Focant et al., 2013). In summary, our findings show that astrocytes use a balance of Arp2/3 activation and inhibition through N-WASP, WAVE2 and PICK1 to maintain and modulate their morphology. This mechanism underlies the morphological changes associated with astrogliosis that occur in response to CNS injury.

## Materials and Methods

### Ethical approval

Animal care and experimental procedures were conducted in accordance with British animal protection legislation and experimental protocols approved by the British National Committee for Ethics in Animal Research.

### Cell culture

Cortical astrocytes were isolated from brains of postnatal rats (P2). Cerebral cortices were isolated, mechanically dissociated in HBSS and trypsinized (Invitrogen, Paisley, UK) for 15 min at 37°C. Afterwards, trypsin (Invitrogen) was inhibited by triturating cells in complete Dulbecco's modified Eagle's medium (DMEM) (Lonza, Slough, UK) with 10% FBS. Cell suspensions were subsequently plated into T75 flasks (Greiner-BioOne, Frickenhausen, Germany), coated with 0.025% collagen and 100 µg/ml poly-L-lysine. Astrocytes were cultivated till confluency and, subsequently, plated for imaging or biochemical experiments. Microglia were erased by treating astrocytes cultures at high density for 90 min with 60 mM L-leucine-methylester in complete medium (Hamby et al., 2006). Contaminations by other cell types were prevented by plating astrocytes onto uncoated glass coverslips (Silva et al., 1998) or glass-bottomed dishes (MaTek Corporation, Ashland, MA, US). For western blotting, L-leucine-methylester-treated astrocytes were plated onto plastic six-well plates (Greiner-BioOne), coated with 0.025% collagen and 100 µg/ml poly-L-lysine. Acute cortical slices (P14) were obtained as described previously (Perugini et al., 2012). Cortical and hippocampal rat neurons were prepared and cultured as published previously (Nakamura et al., 2011). Murine embryonic fibroblasts were kindly provided by Chun Guo (University of Bristol, Bristol, UK). MDA-MB-231 and HEK293T cells were cultivated in complete DMEM medium with 10% FBS on uncoated plastic dishes.

### Pharmacological treatments

Stellation of dissociated astrocytes was induced by 2 h of serum starvation in DMEM medium and subsequent incubation with 10 µM forskolin in DMEM for 2 h. Stellated astrocytes were then used for destellation experiments with 75 µM CK-548 in DMEM. The following reagents were used in conjunction with CK-548 for 2 h on previously stellated astrocytes: SMIFH2 (75 µM), Blebbistatin (100 µM) and Y-27632 (25 µM). In all experiments, DMSO served as vehicle control. All reagents were purchased from Sigma-Aldrich (St Louis, MO, US). Acute slices were placed in oxygenated artificial cerebrospinal fluid (aCSF) and incubated for 2 h with either CK-548 or vehicle control.

### siRNAs

siRNAs were transfected into astrocytes using Lipofectamine RNAiMax: astrocytes were transferred into serum-free Opti-MEM (Invitrogen) and transfected with siRNAs, as published previously (Benfenati et al., 2011). After 4 h of incubation, serum was added to transfected cells, obtaining a final concentration of 10% FBS. On the following day, Opti-MEM was replaced with DMEM-based cultivation medium, as described above. To increase the transfection efficiency, this procedure was repeated 3 days after the initial transfection. siRNAs directed against Arp3 (Korobova and Svitkina, 2008) and WAVE2 (Danson et al., 2007) were purchased from Dharmacon (Lafayette, CO, US). The N-WASP-specific siRNA, based on a previously published shRNA (Kovacs et al., 2011), was purchased from Sigma-Aldrich. The PICK1-specific siRNA is based on a validated shRNA sequence (Citri et al., 2010) and was purchased from Sigma-Aldrich. The unspecific siRNA (5′-AGGUAGUGUAAUCGCCUUGTT-3′) was purchased from Eurofins MWG (Regensburg, Germany).

### Plasmids and transfection

Constructs encoding for GFP fusion proteins of either wild-type N-WASP (pEGFP-C1-N-WASP WT, Lommel et al., 2001) or the constitutively active N-WASP mutant (pEGFP-C1-N-WASP Δ227–267, Stamm et al., 2005) were a generous gift by Theresia Stradal (University of Münster, Germany). pEGFP-C2 served as negative control. Plasmid DNA was transfected into astrocytes by using Lipofectamine LTX (Invitrogen), as described previously (Benfenati et al., 2011), with the above mentioned medium changes used for siRNA transfection.

### Antibodies and reagents

Mouse anti-β-actin AC-15 (Sigma-Aldrich, IF 1∶1000), mouse anti-Arp3 [Sigma-Aldrich; 1∶200 for immunofluorescence (IF), 1∶1000 for western blot (WB)], mouse anti-S100β SH-B1 [Sigma-Aldrich; 1∶1000 for immunohistochemistry (IHC)], guinea pig anti-GFAP (Synaptic Systems, Göttingen, Germany; IF 1∶500, IHC 1∶400), rabbit anti-WAVE2 D2C8 (Cell Signaling, Beverly, MA; WB 1∶1000), rabbit anti-N-WASP #4848 (Cell Signaling; WB 1∶1000), mouse anti-GAPDH 6C5 (Abcam, Cambridge, UK; WB 1∶20,000), rabbit anti-RhoA (Cell Signaling; WB 1∶1000), mouse anti-Rac1 610651 (BD Biosciences, Franklin Lakes, NW; WB 1∶1000), mouse anti-PICK1 L20/8 (Antibodies Incorporated, Davis, CA; WB 1∶1000), chicken anti-PICK1 NBP1-42829 (Novus Biological, Littleton, CO; IF 1∶300). Horseradish peroxidase (HRP)-conjugated secondary antibodies were purchased from Millipore. Cyanine dye conjugated and cross-absorbed secondary antibodies (IgGs) were purchased from Stratech (Newmarket, UK). Filamentous actin was labeled with Alexa Fluor (Alexa 546, Alexa 633)-conjugated phalloidin (Invitrogen). DNA staining was carried out using Hoechst 33258 (Invitrogen).

### Immunocytochemistry

Indirect immunofluorescence with formaldehyde fixation only was performed as described previously (Murk et al., 2009). Immunofluorescence for PICK1 required methanol fixation. Astrocytes were fixed and permeabilized in –20°C cold methanol for 3 min. Subsequent immunostaining was carried out, as described in the mentioned reference. After methanol fixations cell morphology was visualized by antibody staining for β-actin. Cells were imaged on either LSM 510 Meta (Zeiss, Jena, Germany) or Leica SP5-II (Leica, Heidelberg, Germany) confocal microscopes using a 40× objective (1.3 NA) and a multitrack mode to acquire individual channels separately. Image processing was performed in ImageJ.

### Deep tissue Immunohistochemistry and tissue clearance

A recently published protocol to clear brain tissue (Hama et al., 2011) was modified to allow its combination with whole-mount immunohistochemistry. After the above mentioned pharmacological treatments, 400-µm thick slices from the rat motor cortex (P14) were fixed for 1 h in 4% (w/v) formaldehyde in PBS at 4°C. After three washes in PBS, slices were permeabilized in 2% (v/v) Triton-X100 in PBS overnight. After three subsequent washes in PBS, slices were incubated with 20% BSA in PBS for 1 h, then incubated with primary antibodies in PBS with 0.1% Tween-20 (PBS-T) for 36 h. After repetitive washes with PBS and a subsequent incubation in 20% BSA in PBS, slices were incubated with species-cross-absorbed secondary antibodies in PBS-T for a further 36 h. Nuclei were stained by the application of 0.5 µg/ml Hoechst 33258 (Invitrogen) for 5 min at room temperature. To avoid the denaturation of antibodies by the Scale reagent, stained slices underwent an additional fixation with 4% formaldehyde in PBS for 1 h at 4°C. Afterwards, fixed cultures were incubated in ScaleA2 for 4 days at 4°C, followed by another 2 days with ScaleB4. Finally, slices were mounted and imaged with a single photon confocal microscope (Leica SP5, Leica) using a standard 63× immersion objective (NA 1.7). Astrocytes were acquired in multitrack mode as *z*-stacks with 1-µm thick confocal sections.

### Live-cell imaging

The phase-contrast supplementary material movies were recorded in Phenol-Red-free DMEM at 37°C and 5% CO_2_ with a Leica AS-MDW live-cell imaging workstation (Leica) equipped with Roper CoolSnap HQ 12-bit monochrome CCD camera, Maerzhaeuser scanning stage and environmental control chamber (Solent, Segensworth, UK) with long-term temperature control and CO_2_ enrichment. For live imaging, a 40× objective (1.3 NA) was used. Images were acquired every minute in a multi-acquisition mode and assembled into movies using Volocity software (Perkin-Elmer, Waltham, MA, US).

### Oxygen/Glucose deprivation (OGD)

OGD was performed within a MACS-VA500 anaerobic workstation (Don Whitley Scientific Limited, Shipley, UK) supplemented with 95% N_2_ and 5% CO_2_ at 37°C for 5 or 20 min. Astrocytes were washed twice with deoxygenated serum-free DMEM without glucose (Invitrogen) and were then maintained in this medium under anaerobic conditions. Immediately after OGD, cells were washed once with 1× PBS and fixed for 20 min with 4% formaldehyde followed by phalloidin staining. In some experiments, reperfusion with oxygenated and glucose-containing DMEM without serum followed OGD treatments: astrocytes were transferred from the anaerobic workstation into a humidified and air-supplemented incubator and incubated at 37°C and 5% CO_2_ for 3 h.

### GTPase activation assays

Isolation of active GTPases from lysates by pulldown assays was performed as described previously (Pellegrin and Mellor, 2008).

### Morphological analysis

Dissociated astrocytes were categorized into polygonal and stellate astrocytes by calculating the ratio of cell outlines and total cell areas with Fiji software (Schindelin et al., 2012). Low-density cultures were used and single stellate astrocytes were filament-traced by Imaris to allocate processes to individual cells (Bitplane, Zurich, Switzerland) before cropping in Fiji. Astrocytes were defined as polygonal if they had a cell-outline:cell-area ratio <0.2. Changes in the proportion of polygonal astrocytes upon different treatments were statistically determined in the same manner as in previous studies (Burgos et al., 2007; Favero and Mandell, 2007). Complexities of astrocyte processes, without taking the cell body into account, were measured by Sholl analysis using the filament tracer of the Imaris software. To study morphological properties of astrocytes within tissue, only astrocytes in layer 2 or 3 of the motor cortex and at a depth of 15- to 65-µm within the tissue were taken into account. Signal-to-noise ratio was improved by using the ‘despeckle’ function in Fiji. Files were then exported to Imaris software and further processed by 3D cropping of individual cells, baseline thresholding and gausian filtering. Astrocyte morphology was determined by using the Imaris filament tracer, whereas GFAP-positive processes of individual astrocytes were traced first and used as main tracks to determine smaller and branched processes positive for S100β.

### Statistical analyses

To determine statistical significance in results obtained from GTPase activation assays and measurements on proportions of polygonal astrocytes, an unpaired Student's *t*-test with Welch's correction was performed. For multiple analyzes, data were analyzed by using ANOVA followed by Bonferroni's corrections. Sholl analyzes were assessed using unpaired Student's *t*-tests and the Sidak–Bonferroni method. On all graphs, error bars are standard deviations. *P*-values are defined as followed: *P<0.05, **P<0.005, ***P<0.0005. Statistical tests were calculated by using Excel 2010 (Microsoft) and Graphpad Prism 6 (Graphpad Software, San Diego, CA).

## Supplementary Material

Supplementary Material
