# Supplementary material for: The antagonistic modulation of Arp2/3 activity by N-WASP, WAVE2 and PICK1 defines dynamic changes in astrocyte morphology
Source: J Cell Sci. 2013 Sep 1;126(17):3873–83. doi: 10.1242/jcs.125146 (PMC3757329; doi:10.1242/jcs.125146)
Supplement: Supplementary Material [file supp_126_17_3873__index.html]

The antagonistic modulation of Arp2/3 activity by N-WASP, WAVE2 and PICK1 defines dynamic changes in astrocyte morphology — Supplementary Material 

# The antagonistic modulation of Arp2/3 activity by N-WASP, WAVE2 and PICK1 defines dynamic changes in astrocyte morphology

## JCS125146 Supplementary Material

**Files in this Data Supplement:**

- **Supplementary Material PDF**
