# Supplementary material for: The antagonistic modulation of Arp2/3 activity by N-WASP, WAVE2 and PICK1 defines dynamic changes in astrocyte morphology
Source: J Cell Sci. 2013 Sep 1;126(17):3873–83. doi: 10.1242/jcs.125146 (PMC3757329; doi:10.1242/jcs.125146)
Supplement: Supplementary Material [file supp_126.17.3873_JCS125146.pdf]

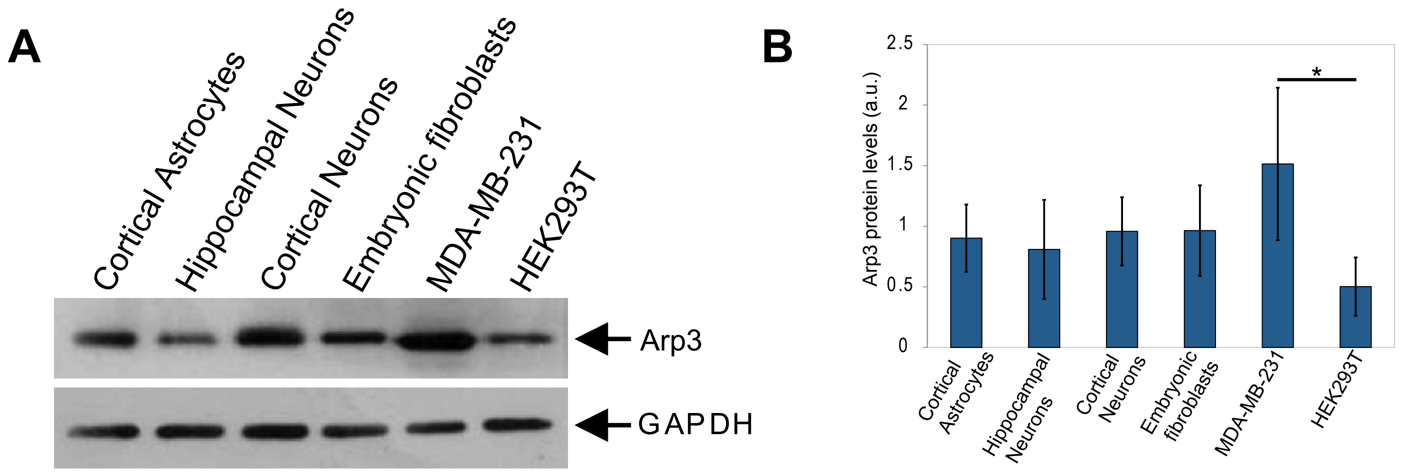

Fig. S1. Levels of Arp3 do not significantly differ in astrocytes, compared to other cell types. (A) Lysates of cortical astrocytes, hippocampal neurons, cortical neurons, primary embryonic fibroblasts, MDA-MB-231 cancer cells and HEK293T cells were analysed for the expression levels of Arp3 and GAPDH by Western blotting. (B) Quantification of Arp3 levels in cells displayed in (A). n=5-6, *$p < 0.05$ (ANOVA followed by Bonferroni`s correction)

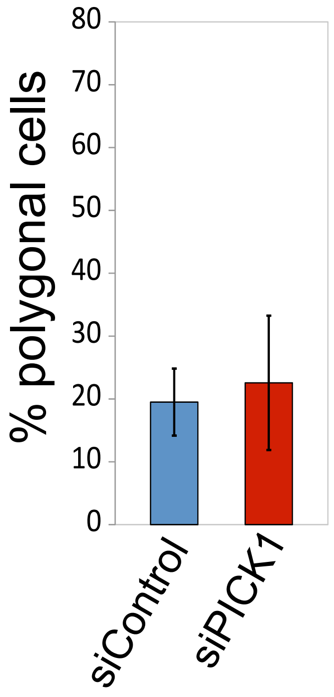

Fig. S2. Quantification of polygonal astrocytes after PICK1 depletion and forskolin treatment. Quantification of the proportion of polygonal astrocytes after PICK1 depletion followed by serum starvation and forskolin treatment, as shown in Figure 4L. *$p < 0.05$ (unpaired t-test).

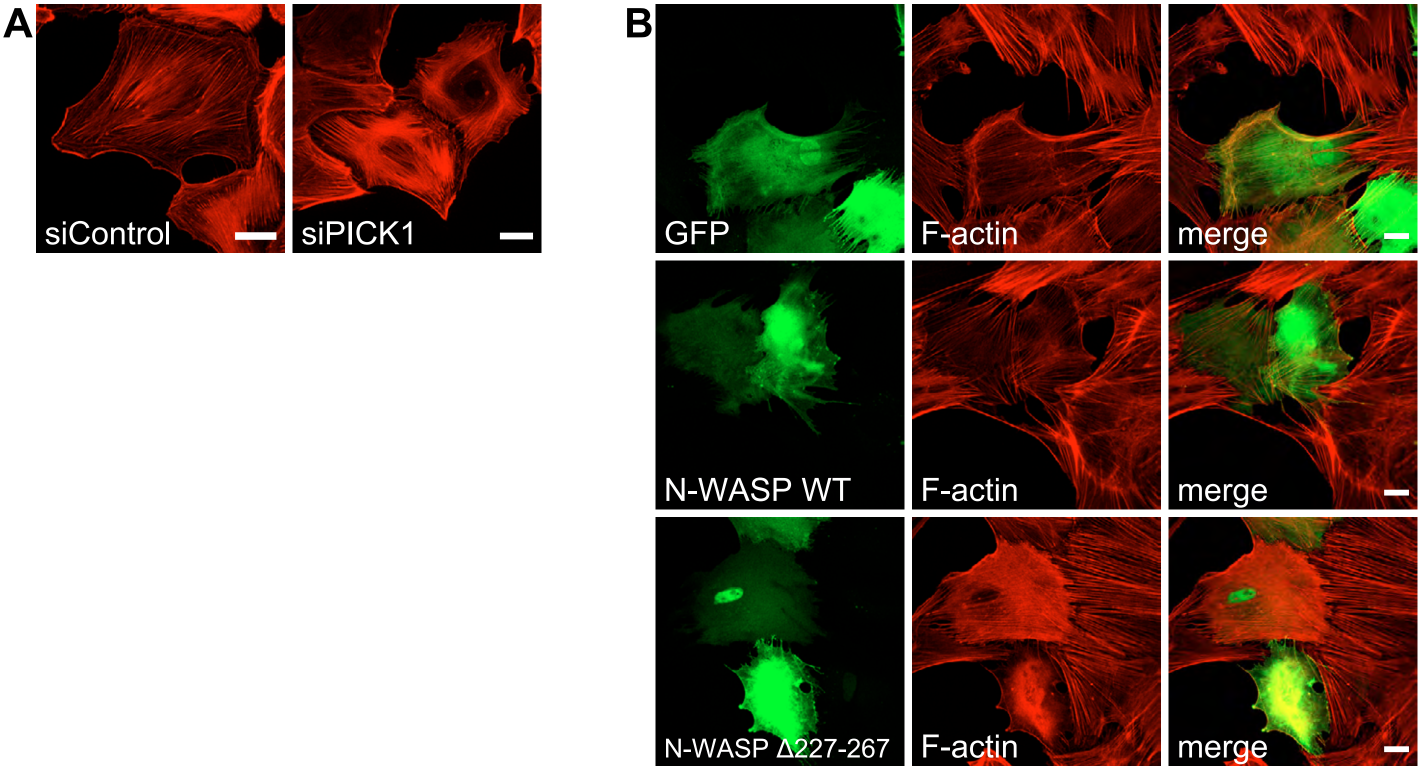

Fig. S3. Study of PICK1 depletion and overexpressed N-WASP variants in polygonal astrocytes. (A) Confocal images of polygonal astrocytes transfected with control (siControl) and PICK1 (siPICK1) specific siRNAs, followed by fixation and phalloidin staining. Bars: 10 µm. (B) Confocal images of polygonal astrocytes transfected with GFP, GFP-N-WASP WT or constitutively active GFP-N-WASP-Δ227-267, stained with phalloidin. Bars: 10 µm.

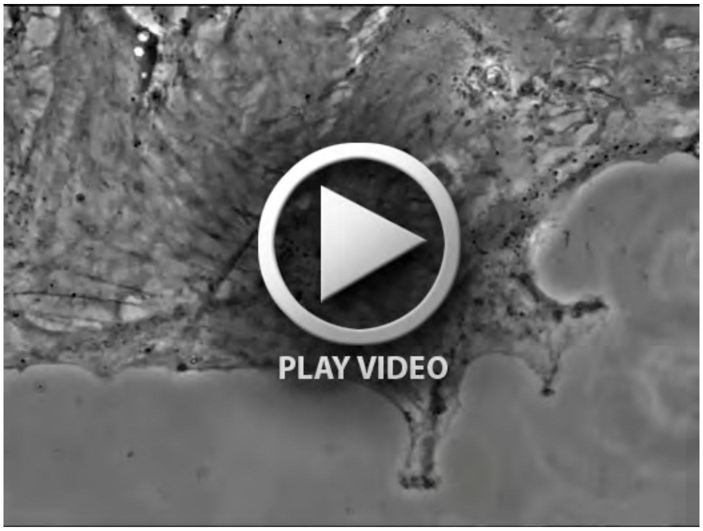

Movie 1. Time-lapse video of control astrocytes treated with forskolin. Serum starved astrocytes were treated with forskolin in basal medium and observed under phase-contrast for one hour. Video begins immediately after adding forskolin.

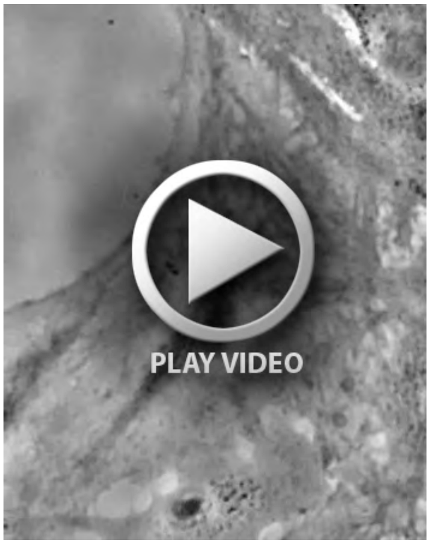

Movie 2. Time-lapse video of astrocytes simultaneously treated with forskolin and CK-548. As in Movie 1, cells were previously serum-starved and then recorded for one hour immediately after adding CK-548 and forskolin.

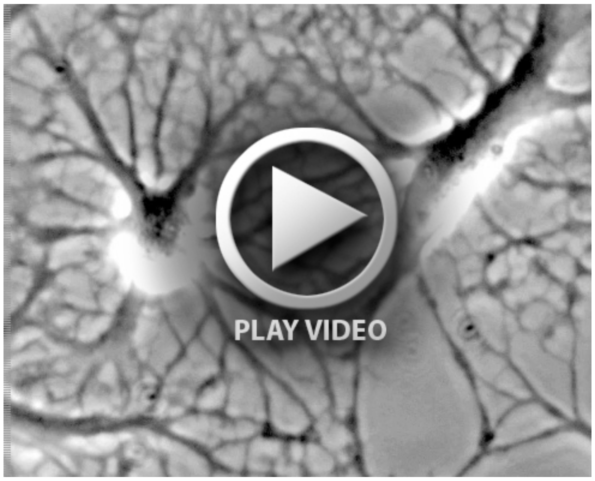

Movie 3. Time-lapse video of astrocytes after forskolin washout in DMSO containing medium. Astrocytes were stellated as shown in Movie 1 and subsequently incubated in DMSO supplemented serum-free medium. Video was recorded for 1 hour.

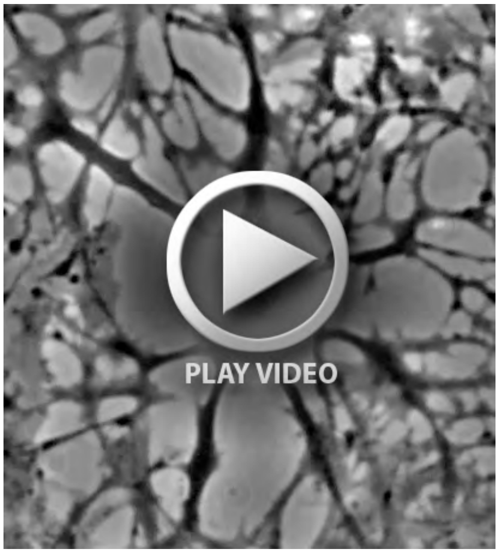

Movie 4. Time-lapse video at high magnification on an individual astrocyte incubated with CK-548 after forskolin removal. Time-lapse recording of a stellate astrocyte at high magnification treated for one hour with CK-548 after forskolin washout. Note the filopodia formation and membrane blebbing which lead to cell expansion. Video recording started right after addition of CK-548.

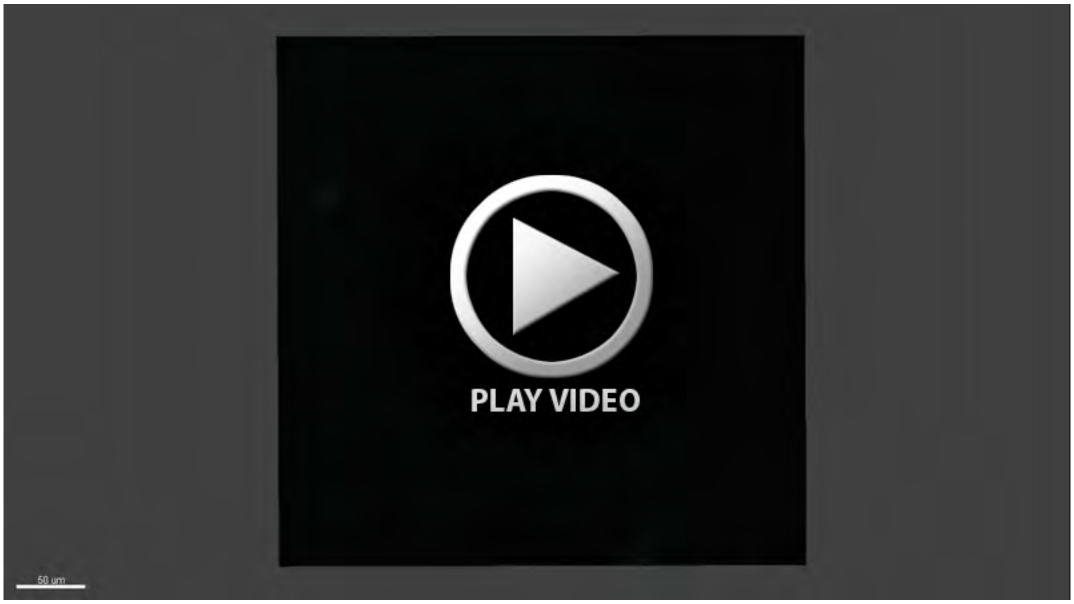

Movie 5. Visualizing of antibody labeled astrocytes in brain slices after tissue clearance. Series of X-Y images of 3D reconstruction data acquired from an acute cortical slice after tissue clearance and staining for GFAP (green) and DNA (blue). Confocal sections (2 µm thickness) were acquired with a standard single photon microscope and 40x objective (NA 1.3) to a depth of 120 µm. In this example, settings for laser power and detector gain were kept constant. Note that beyond the areas traumatized by slicing, typical astrocytes as well as blood vessels ensheathed by astrocytic endfeet are visible.

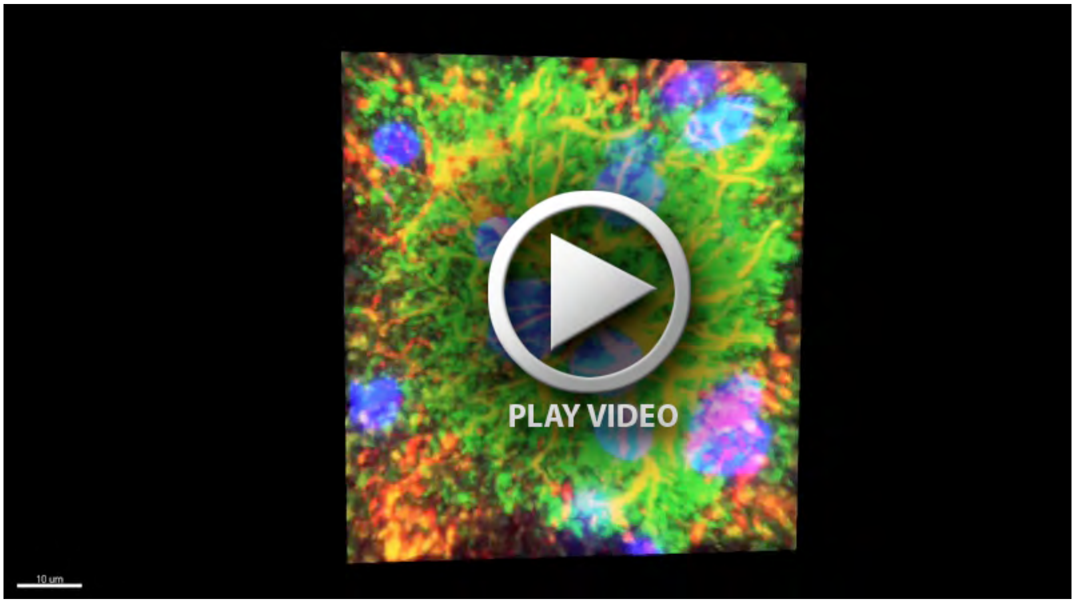

Movie 6. Insight into a 3D reconstruction of an immunohistochemically labeled astrocyte. To demonstrate the detailed visualization of astrocyte morphology, a subset of a 3D reconstructed astrocyte is shown as rotating animation. Astrocytic main processes are labeled with GFAP specific antibody (red), the complex fine structures with S100β specific antibody (green), DNA by Hoechst 33258 staining (blue).
